# Periodicity and Intermittency of Disease

**Published:** 1856-06

**Authors:** Abrm. Livezey

**Affiliations:** Lumberville, Pa.


### ON PERIODICITY, INTERMITTENCY OR REMITTENCY OF DISEASE.

BY ABRM. LIVEZEY, A. M., M. D., LUMBERVILLE, PA.

Having laid down the general principles of, what we hold to be, a sound medical philosophy, in the previous article, which are to guide us in the investigation of diseased action, as it is manifested in the *corpus humanum*, we are prepared to consider the nature and treatment of diseases generally: And I hope to show, *seriatim*, that all diseases have something in common—have each some principle of continuity, which amid all their apparent diversity, establishes a unity of type, as characterized by periodicity and intermittency.

The idiopathic or "essential" fevers being of such frequent occurrence, and so destructive to human life, according to the estimates of Sydenham, Van Swieten and Eberle, it becomes a source of interesting inquiry, firstly, to determine accurately their nature and best mode of treatment.

The great error in regard to intermittent fever, or ague, is to attribute it mainly to the effects of miasm—a peculiar exhalation from soils—which pervades, and after a period of incubation, poisons the system; whilst there are in nature a thousand causes instead of one. For, every well read medical man (to throw aside his own observations as naught) knows full well, that ague is caused by a passion, by fear, by mechanical, medical and chemical means,—by change of habits, depression of spirits, loss of fortune, bankruptcy, etc., as well as by the fabled miasm.

For if "miasm" be the "cause" only, what becomes of it for hours, or even whole days together, when the patient enjoys his usual health in the intermission?

Or how is it that spiders' web, snuff of candle-wicks, a passion or a charm even, destroys its fell potency? But wherefore this intermission, be the cause what it may? Why does the paroxysm recur again and again? These questions can only be answered by admitting (a self-evident truth, by the way) that periodicity is a law of all animal movement in disease as well as in health—like all the immutable laws of nature—there are tidal fluxes and refluxes—alternate attraction and repulsion of every organ and atom of the living body. In nature we have spring time and harvest, summer and winter, heat and cold, day and night,—periodical return of comets, of tides, of the fish of the sea, of the fowls of the air,—all are regulated by fixed, periodical laws. What more can, or need be said?

An error equally great as to the nature of ague is arrived at, in post mortem examinations: Congestion of the internal viscera is found, and hence "congestion, or inflammation or something like it" is considered the nature of ague,—the *end* being taken for the *beginning*. For the internal organs are equally congested in those who die in a passion, or in the shock of fear, or even in a fainting fit, caused, too, by loss of blood!

Hence ague, like fear, faint or a passion, exerts its force upon the brain and nerves, and to these we should look for its *nature*, and upon no other theory can its cure, whether by quinine or otherwise, be explained.

The truth is this: that ague is curable by any thing, agent or circumstance, which acts in a novel manner on every atom of the living brain and nerves, and through these on every organ and atom of the body. Thus the curative effect ot any of these agents consists in their producing an order of movement of the atoms of the brain and nerves, of a contrary kind to the order of movement which prevails in them during the continuance of the fever for which it is given.

The application of the *intermission period* to other diseases, is as numerous as are the diseases recognized by the schools. Let me instance a few examples:—

Mrs. S. had a violent pain in her left side for 30 minutes at a time, which recurred about every two hours. A full inspiration augmented the pain, and she had been treated, under the supposition of an highly irritated or inflamed diaphragm, with cups, blisters, neutral mixture, Dover's powders, etc., without relief. The following, given at the subsidence of a paroxysm, prevented its return:

℞. Quin. Sulph. gr. x.
Ferri Subcarb. " x.
Zingib. pulv. " v.

Several such attacks has she had subsequently and could find relief only from prescriptions, which contained quinine.

Mr. L. had been afflicted with a violent pain in the right hypochondriac region "by spells" for some days. Under a fanciful supposition that it was a hepatitis, or a spasm ot the biliary ducts, cupping, pil. hydrarg, etc., had been resorted to, in vain. A few doses of quin. sulph. and fer. carb. given in the absence of the pain, cured him.

Mr. P. complained of great dulness of hearing and ringing through one side of the head, of a very distressing nature, in the afternoon and evening for 14 years.—Had consulted many physicians, and after submitting to the routine of cupping, leeching, blistering, pustulation, etc., with no success, the disease had been pronounced to be *organic* and irremediable. The following soon cured him :

℞ Acid. hydrocyan. Medicin. gtt. ii–iii.

Tinct. cinchoniæ ℥i. Ter die.

Mrs. M. was troubled "off and on" with some erysipeloid humor, or inflammation of the ear, for years; had *doctored* much and spent much of her "substance," but "grew nothing better;" was cured permanently by taking Liq. potass. arsenit. gtt. vi. ad x. ter die for a short time. Mr. J. was laboring under pneumonia—fever, cough, crepitant rale passing into the mucous, general distress; high fever, thirst, dyspnœa, harassing cough ensued every day about 11 o'clock, which continued till late in the night, when the symptoms relaxed by copious perspiration, etc. Ten grs. quin. sulph. given at 10 o'clock in the morning for three successive mornings dispelled every unpleasant symptom. But why anticipate more? These cases are sufficiently numerous and various to show the profession under what variety of forms we have intermittent fever; and also by these, are we taught the correct principle of treatment.

But when, from any cause, the intermissions, or immunity from suffering are greatly shortened, and instead of extending to a day or days, they are only an hour or two in duration, the disease is then nosologically designated Remittent fever,—a fever greatly modified or influenced by place, season, temperature, habit, etc., and consequently its developments, as to intensity, greatly differing at different times and in various localities. Hence the various names for these several modifications, arising from the incidentals named, as Bilious, Typhoid, Typhus, Yellow, Nervous fever, etc., etc., and yet it must be admitted that these several forms of "essential fevers," (Wood,) with all their diversity and aggravation of symptoms, are only modifications and aggravations of the phases of a simple intermittent or remittent fever.

Dr. John Clark, an eminent physician, in "Observations on Fevers, London, 1792," thus writes: "After several years attention to the symptoms and nature of fevers, as they have occurred in different climates, I freely confess that I have not been able to follow authors through their numerous divisions and subdivisions." Again, "I am fully convinced that although many varieties happen, according to constitution, season, situation and climate, yet everywhere fever is essentially the same, or, in other words, consists of one GENUS."

From the incidentals above named, remittent fever varies, and yet, as in all the essential fevers, the *common causation* doctrine holds good, whether the fever be nicknamed remittent, typhoid, typhus, yellow fever, adynamic, etc. That such is the fact—that we have as great differences in the forms and attacks of bilious remittent fever, is fully and satisfactorily illustrated by the nature of scarlatina, and the different characters which it assumes: For instance, we have the simple, the anginose, the sine exanthematous and malignant forms, side by side, in different visitations, which are very various in attack, form, etc., and yet it is all scarlatina—all from one source, and we do not think of calling it anything else because it presents these different characters. So with "fevers," all are remittent, all the same thing at last. And the individual predisposition, the location and the intensity of the poisonous cause account for morbid and anatomical differences. And in all forms of fever, sooner or later and more or less complete, comes a *remission period*,—a fact to be kept in mind—a period to be watched for, because all sound, rational treatment must be based upon it.

"At the commencement," says Prof. Wood, of Philadelphia, "there may be two or more regular paroxysms, as in intermittents, afterwards the paroxysms may recur regularly without the chill or the perspiration." * * * "The duration of the chill varies from a few minutes to an hour or more." * * * "These symptoms (headache, thirst, heat of skin, etc.,) continue without abatement usually from six to eighteen hours, after which they begin to relax with the appearance of moisture about the neck and face." Here is the "remission," which continues for "two or three hours" or a "whole day," when another paroxysm of fever takes place, etc. How plain is the indication here! How easily are such attacks of fever arrested *in limine!* How blind are physicians to the importance and value of these remissional periods! Prof. Wood, however, profits not by them, but suffers the disease to continue—the paroxysms to recur again and again—under the administration of calomel, refrigerant diaphoretics, antimonials, effervescing draughts, etc., all of which, however, do not prevent the tongue becoming "thickly covered with a yellowish white coat, and often brown and black,"—"tenderness upon pressure in the epigastrium,"—"nausea and vomiting of a yellowish green color," "stools dark colored and offensive," etc., etc., symptoms and conditions more from the effects of the medicine advised, than from the natural course of the disease.

Now let it be remembered that paroxysm and remission are the characteristics of every development of disease; and if this great fact be overlooked and mis-improved, the treatment is of no good avail, as is evidenced by the fact, that there is a continually increasing prostration and exhaustion; a highly irritated condition of the stomach and intestines; tympanitis and ulceration; subsultus, delirium, typhoid or enteric fever; and not unfrequently death, if not averted by the administration of "turpentine" or "quinine" in the last stages of the disease.

What are the indications, and how should this *fever* be treated? The first and important indication is to procure a decided remission, to be attained by an emetic of ipecac., or, if "bilious symptoms" be present, such as yellow-coated tongue, bitter taste, fulness and distress in the epigastric and right hypochondriac regions, then it is better to conjoin calomel thus:

℞. Hydrarg. chlorid. mit. }

Pulv. rad. ipecac. } aa grs. xv.

Misce et divide in pulv. iii.

One to be taken every hour. Or in cases of nausea, etc., jalep may be substituted for ipecac.; the cathartic action of either to be assisted, if the case requires it, by senna and jalep, the compound powder of jalep or senna and salts. Speedy relief of all the prominent symptoms generally follows and the skin becomes relaxed and moist for some time. To augment the latter and to get a full febrifuge effect, spiritus mindereri, sweet spirits of nitre, tincture of aconite (folia) with or without the addition of tartar emetic to either of these, or infusions of ipecac., or lobelia even, every 15 or 30 minutes, secures a very copious perspiration and great relief to the patient. Now is the time to administer the sulphate of quinine, as recommended by Prof. T. D. Mitchell:

℞. Quin. sulph. gr. x. }

Pulv. zingib. " v. } Misce et. divid. in iv parts æquales.

One to be taken in sugared water every half hour.

The appearance of moisture is to be taken as evidence of actual remission. "It was deemed an unwise course," says Prof. Mitchell, in speaking of the treatment of epidemic bilious remittent fever, as it prevailed in many parts of Pennsylvania, during a period extending from 1823 to 1827, "to wait for total absence of heat, or in other words, for an *intermission*, since that is no part of a *remittent*. The moment I saw an evident abatement, there was remission enough to warrant the use of the antiperiodic power of the salt of quinine. My theory and the practice, and the issue, all harmonized here most perfectly, for I never in my whole medical history have witnessed as perfect a triumph of medicine over disease as I saw for weeks and months in this procedure in the treatment of *bilious remittents*." (*Therapeutics*, p. 124 & 125.)

Dr. Jno. Clark in the treatise on fevers, before alluded to, pursued a similar course in the Newcastle fever in 1777: viz. Mild emetic, mild purgative, next induce perspiration and then administer bark. He condemns venesection and says he never lost a patient from the omission. Of 64 patients treated by Dr. C. but two died.

Dr. Hume writes from the coast of Africa, that, "we had to contend with a fierce enemy—bilious remittent fever—as it rages between the tropics. But of 66 cases, we had not a death. This was effected not by bleeding, leeching, cupping, salivating, physicing, drugging—but by quinine in large doses. This knocks the disease on the head at once, and the patient recovers, not a blanched and wretched remnant of humanity, but in a few days well and useful as ever."

Dr. Brett, physician to the Fever hospital, Liverpool, says that of "700 cases treated with emetics, laxatives and quinine, the deaths were 1 in 10½; formerly, under the ordinary treatment, 1 in 6."

Dr. Lacombe, writes thus from Port Cabello, Venezuela: "We seldom lose any, whether from intermittent, remittent or typhoid. A favorable modification is obtained by emetics, (i. e. remission, more or less complete) and then quinine or piperine effects a cure. When bleeding was resorted to, and which was formerly the general practice, the average number of deaths were from 20 to 30 per cent.; now only 2½ to 4."

By the adoption of the treatment above, the paroxysmal repetitions are at once averted, or very greatly subdued in their intensity, and local determinations are prevented; for they are seldom present in the beginning of the fever, (Wood) but only arise from repeated recurrence of the paroxysms.—*Western Lancet.*

